# Effect of Various Hydration Strategies on Work Intensity and Selected Physiological Indices in Young Male Athletes during Prolonged Physical Exercise at High Ambient Temperatures

**DOI:** 10.3390/jcm13040982

**Published:** 2024-02-08

**Authors:** Tomasz Pałka, Łukasz Rydzik, Piotr Michał Koteja, Anna Piotrowska, Małgorzata Bagińska, Tadeusz Ambroży, Boryana Angelova-Igova, Norollah Javdaneh, Szczepan Wiecha, Aleksandra Filip-Stachnik, Łukasz Tota

**Affiliations:** 1 Department of Physiology and Biochemistry, Faculty of Physical Education and Sport, University of Physical Education, 31-571 Kraków, Poland; wfpalka@wp.pl (T.P.); lukasz.tota@awf.krakow.pl (Ł.T.); 2 Institute of Sports Sciences, University of Physical Education, 31-571 Kraków, Poland; piotr.koteja@awf.krakow.pl (P.M.K.); tadeusz.ambrozy@awf.krakow.pl (T.A.); 3 Department of Chemistry and Biochemistry, Faculty of Physiotherapy, University of Physical Education, 31-571 Kraków, Poland; anna.piotrowska@awf.krakow.pl; 4 Institute of Biomedical Sciences, Department of Physiology and Biochemistry, University of Physical Education, 31-571 Kraków, Poland; malgorzata.baginska@doctoral.awf.krakow.pl; 5 National Sports Academy Vassil Levski, Philosophy and Sociology of Sport, 1700 Sophia, Bulgaria; igovab@gmail.com; 6 Department of Biomechanics and Sports Injuries, Kharazmi University of Tehran, Tehran 14911-15719, Iran; njavdaneh68@gmail.com; 7 Department of Physical Education and Health in Biala Podlaska, Faculty in Biala Podlaska, Jozef Pilsudski University of Physical Education, 00-968 Warsaw, Poland; 8 Department of Sports Medicine and Human Nutrition, Institute of Biomedical Sciences, University of Physical Education, 31-571 Kraków, Poland; aleksandra.filip@awf.krakow.pl

**Keywords:** thermoregulation, ambient temperature, hydration, physical exercise

## Abstract

**Background**: In high temperatures, adequate hydration is vital for sustained physical exercise. This study explores the effect of three hydration strategies on physiological indices and work intensity. **Methods**: The research involved 12 healthy males who engaged in three test series, each separated by a one-week interval. During the trials, participants underwent a 120 min cycling session in a thermal climate chamber (temperature: 31 ± 2 °C, humidity: 60 ± 3%, air movement: <1 m/s). Measurements of rectal temperature (Tre) and heart rate (HR), and assessment of subjective workload perception, and thermal comfort were made both before and during the exercise. The computation of the physical strain index (PSI) relied on Tre and HR values. Three hydration strategies (isotonic drink, water, and no hydration) were administered before, during, and after the exercise. **Results**: Regardless of the hydration strategy, the participants’ mean body mass decreased as a result of the exercise. Statistically significant differences in HR were observed between the no-hydration and water groups (*p* < 0.036). The mean PSI values significantly varied between hydration strategies, with the no hydration group exhibiting a higher PSI compared to the isotonic drink or water groups (*p* < 0.001). **Conclusions**: All hydration strategies contribute to thermoregulatory processes and mitigate the rise in internal body temperature during sustained physical exercise in elevated ambient temperatures.

## 1. Introduction

During sustained endurance physical exercise at elevated ambient temperatures, an adequate supply of nutrients, including fluids, is extremely important. Insufficient amounts of nutrients can cause numerous adverse changes, including impaired exercise capacity due to reduced plasma and circulating blood volume [1,2]. Adequate hydration during exercise contributes to the maintenance of high-level physical capacity and cognitive function and supports body thermoregulation [3,4,5]. People who engage in sustained physical activity increase the excretion of water and electrolytes through sweat, which can affect heart and muscle function [6,7]. Depending on the type of activity, weather conditions, sex, or individual circumstances, the rate of perspiration during physical exercise varies between 0.3 and 2.4 L/h [8,9,10]. To counteract the negative effects of dehydration, fluids should be replenished during exercise in such a way that no more than 2% of body mass is lost. This level of dehydration results in a 10–20% reduction in maximal aerobic capacity [11,12].

The physical exercise undertaken at elevated ambient temperatures constitutes a specific effort. It induces increased water loss with sweat, which can lead to hypovolemia and, consequently, a much higher cardiovascular load, enhanced glycogen consumption, and central nervous system dysfunction [7,8]. Continued work under such conditions leads to water and electrolyte disturbances, as well as thermal balance and intracorporeal homeostasis disruption; these aggravate the physiological and biochemical changes associated with the physical work undertaken [13,14]. Ensuring and maintaining an ideal state of hydration during physical activity becomes increasingly challenging depending on the type of sport, the nature of the activity, and the accessibility of the fluids. While optimal hydration is influenced by various factors, it can generally be described as preventing the loss of more than 2–3% of body mass during exercise, while avoiding excessive fluid intake [15]. The survey of the performance of physical work in the heat showed that the best performance during prolonged work was achieved by a fluid intake equal to the volume lost in sweat [16].

The available literature indicates that endurance capacity declines with the magnitude of the fluid deficit and generally increases with a rise in the ambient temperature. Dehydration leads to significantly greater losses of physical endurance during physical effort in warm and hot climates [17,18,19,20].

Dehydration results in a change in the energy metabolism characteristics. Increased glycogen utilization and a higher proportion of anaerobic glucose metabolism in energy production are observed during physical exercise. When exercising under dehydration conditions, increased carbohydrate intake is essential for the correct selection of fluid reserves during effort, with consideration given to the rate of sweating and carbohydrate consumption [21,22]. It is therefore crucial to maintain proper hydration in elevated temperature conditions as it helps preserve the normal function of the cardiovascular and thermoregulatory systems, as well as supporting metabolic processes and effective recovery. The consequences of dehydration may also include impairment in the efficiency of the thermoregulatory mechanisms, with resultant hyperthermia and hypovolemia. Because of the serious medical complications arising from the triad of heat illnesses (heat stroke, heat exhaustion, heat cramps), considerable emphasis is placed on the prevention of these disorders [23,24].

Hydration can be controlled by the oral intake of fluids absorbed with adequate tonic volume and composition. The rehydration strategy should be continued after exercise, as the replacement of lost water and electrolytes is important for maintaining athletic performance. One of the main factors affecting rehydration after exercise is the composition and volume of the liquid consumed [25,26].

During exercise, water and electrolytes are lost as a consequence of thermoregulatory sweating. In some situations, especially if exercise is prolonged, performed at high intensity, and/or in a hot environment, sweat loss can cause excessive water/electrolyte imbalance and impair performance [27]. Therefore, personalized fluid replacement strategies are recommended [28].

Appropriate hydration strategies are based on pre-exercise hydration status and fluid, electrolyte, and substrate needs before, during, and after exercise. Maintaining an optimal state of hydration during exercise depends, for example, on the sport, type of activity, and availability of fluid. Although optimal hydration depends on many factors, it can generally be defined as avoiding both losses greater than 2–3% of body mass and overhydration during exercise [15]. The inappropriate management of fluid intake resulting in hypohydration or hyperhydration can be detrimental to performance and, in some circumstances, increase health risks. The loss of body water during exercise exacerbates physiological and perceptual strain [29] and it is well established that these changes can impair endurance performance, particularly in hot environments, and may increase the risk of exertional heat illness [15]. Although there have been significant advances in the understanding of nutritional requirements for athletes, many gaps still exist in the literature. The science of nutrition remains a constantly evolving and sometimes contradictory complex subject [30].

An effective drinking strategy is essential for optimizing performance during exercise. It is important to consider the athlete’s hydration status before exercise as well as his or her fluid, electrolyte, and substrate needs before, during, and after a workout. This strategy should be tailored to the individual and take into account environmental conditions, competition regulations, and other factors. T therefore, it seems worthwhile to investigate the effects of different hydration strategies during sustained physical effort at elevated ambient temperatures on work intensity and selected physiological indices in young males. The aim of this study was to investigate the effectiveness of different hydration strategies applied during exercise in elevated temperatures and humidity and the impact of these strategies on work intensity ratings and selected physiological indices in young males. It was assumed that isotonic hydration has a greater effect on physiological factors than water hydration.

## 2. Materials and Methods

### 2.1. Group Characteristics

The study involved 12 healthy males aged 20.67 ± 0.98 years with an average level of aerobic capacity (in accordance with the American Heart Association).

The respondents were randomly divided into 6 pairs. During the study, the participants did not use any stimulants, vitamins, or other supplements. Recruitment of participants started on 21 September 2015 and ended on 13 August 2016. The study project was approved by the Ethics Committee at the Regional Medical Chamber in Krakow, Poland (approval No. 42/KBL/OIL/2015) and was supported within the framework of statutory research of the University of Physical Education in Krakow (project No. GRANT 81/MN/INB/2015). Participation in the project was voluntary. The participants were informed about the aims and conduct of the study and provided their written consent to participate. They were also informed that they could immediately withdraw from the study at any time. In accordance with the current standards, the tests were conducted under the supervision of qualified medical personnel and were performed at the Central Research and Development Laboratory, University of Physical Education in Krakow (PN-EN ISO 9001:2015).

### 2.2. Study Design

**Figure 1:**
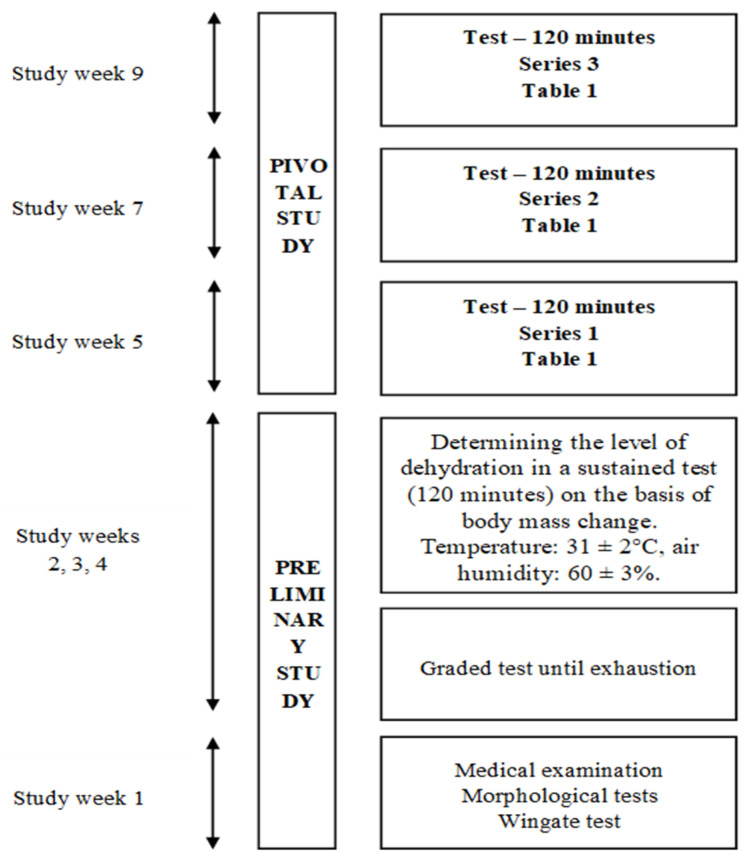
Study design.

The study consisted of 2 main stages, in which distinct tasks were carried out (Figure 1). The first stage, i.e., the preliminary study, included a medical examination, measurement of selected morphological indices, and determination of individual training loads. The second stage was the pivotal study, which involved the training program conducted in a thermal climate chamber. A 3-week training plan was divided into 3 parts, with a 1-week break between particular microcycles. Each microcycle comprised 1 training unit of moderate, constant intensity, with a duration of 120 min. The entire macrocycle consisted of 3 training units. The hydration strategy of each pair of participants was different and depended on the macrocycle.

### 2.3. Preliminary Study

#### 2.3.1. Anthropometric Assessment

Prior to the physiological and biochemical tests, selected somatic indices were evaluated. The indices allowing for the analysis of body composition and body mass were determined by using a JAWON MEDICAL IOI-353 body composition analyzer (certificate: EC0197, Seoul, in South Korea). The 8-electrode bioelectrical impedance technique was applied to assess body composition; lean body mass, body fat mass, body fat percentage, and total body water were determined. Body height was measured with a Martin-type anthropometer (USA) with an accuracy of 0.5 cm. The values of body mass and body height served to calculate the body mass index.

#### 2.3.2. Graded Test

Aerobic capacity was assessed using a direct test method with a gradually increasing load performed on a cycle ergometer (ER 900 D—72475 BIT2, Jaeger, Germany) until exhaustion. Physiological indices were evaluated at the levels of the second ventilatory threshold (VT2) and maximal oxygen uptake (VO2). The exercise load at the VT2 level was monitored in laboratory studies involving elite athletes [31]. To evaluate VT2, we analyzed changes in respiratory indices with increasing work intensity. The criteria for VT2 identification were as follows: (a) the percentage of carbon dioxide in exhaled air reached a maximum value and then decreased; (b) the respiratory quotient of carbon dioxide reached a minimum value and then increased; (c) after exceeding VT2, a large, non-linear increase in lung ventilation was noted. The highest recorded value was considered to be VO_2_ [32].

The test effort was preceded by a 3 min warm-up, during which the participant pedaled at a rate of 60 rpm and an intensity of 110 W. The power was then increased by 30 W every 2 min. The test was performed until refusal to continue due to exhaustion.

During the test, the following indices were recorded by means of an ergospirometer (Cortex MetaLyzer 3B, Leipzig, Germany): respiratory ventilation per minute, the percentage of carbon dioxide in exhaled air, oxygen uptake per minute, carbon dioxide excretion per minute, respiratory quotient, and the respiratory quotient of carbon dioxide. Heart rate was measured with a heart rate monitor (S-610i, Polar, Finland) continuously during the test.

The test was performed at an ambient temperature of 21 ± 0.5 °C and a relative humidity of 40 ± 3%.

#### 2.3.3. Wingate Test

Aerobic capacity was assessed using the direct method of the Wingate test (lower extremities) [33]. The main effort was preceded by a 5 min warm-up on a cycle ergometer (Monark 875E, Sweden) with a load of 1 kg and a pedaling rate of 60 rpm. During the warm-up, three 5 s accelerations were performed at 2, 4, and 5 min. After a 2 min break, the proper test was conducted, in which the participant performed a 30 s maximal physical effort with a load of 8.3% of body mass [34,35,36].

During the test, the following indices were recorded: peak power, mean power, total work, time to obtain peak power, time to maintain peak power and power drop index.

#### 2.3.4. Dehydration Index

Post-exercise water loss was determined with a dehydration index, which constituted the difference between the participant’s pre- and post-exercise body mass (accuracy of measurement: 1 g). The potential volume of urine excreted was taken into account (Sartorius F 1505 electronic scales, DZA, Varberg, Germany). The test lasted for 120 min and was performed in a thermal climate chamber, with an ambient temperature of 31 ± 2 °C, relative humidity of 60 ± 3%, and air movement below 1 m/s.

The ambient temperature and relative air humidity in the thermal climate chamber were controlled with an Ellab electrothermometer (2610 Rødovre. Series CTD85-M 962300 03. Krondalvej, Denmark) and a Harvia thermo-hygrometer (Finland) (accuracy of measurement: ±0.5 °C and ±3%), respectively, and the air movement was measured with a Hill’s caterthermometer.

### 2.4. Pivotal Study

#### 2.4.1. Biochemical Testing

Blood for biochemical analyses was collected from each participant’s ulnar vein by a qualified laboratory diagnostician immediately before and after the stress test, as well as 1, 24, and 48 h after completion of the exercise, under the conditions of a certified laboratory (PN-EN ISO 9001:2015) in accordance with applicable standards. The blood was collected in K2 EDTA tubes. The blood samples were centrifuged (1500 rpm) to separate the serum and, until centrifugation, frozen and stored in ice.

By using an automated method, comprising a Sysmex XN device, hemoglobin concentration and hematocrit, among others, were determined in the whole blood.

Changes in plasma volume (%ΔPV) were estimated with the formula by Dill and Costill [37] as modified by Harisson et al. [38]:

%ΔPV = 100 {(Hb_1_/Hb_2_) · [100 − (HCT_2_ · 0.874)]/[100 − (HCT_1_ · 0.874)]−1}

where Hb_1_ and HCT_1_ represent baseline hemoglobin concentration and hematocrit values, and Hb_2_ and HCT_2_ stand for post-exercise values of these indices; the post-exercise values were adjusted for changes in plasma volume.

#### 2.4.2. Hydration Strategies

The study design assumed the implementation of 3 hydration strategies following the standards adopted by the American College of Sports Medicine [39]. During the exercise, the male participants consumed either an isotonic drink or water at a temperature of 13–15 °C, every 15–20 min, in a volume of 150–300 mL.

Those performing physical exercise without hydration did not receive any fluids. The isotonic drink hydration strategy included the intake of an isotonic drink with an osmolality of 270–330 mOsm/kg water, carbohydrate content of 6–8 g per 100 mL, and sodium (Na^+^) content of 20–50 mmol/L (i.e., 460–1150 mg/L). The water hydration strategy assumed water intake of 120–150% of body mass change.

During the restitution period, after completing the 120 min exercise, the participants stayed at room temperature for 90 min. During the rest, they received isotonic fluids, water, or did not drink any fluids at all. The volume of fluids to be consumed after exercise was determined based on the individual water loss recorded during the preliminary study.

**Figure 2:**
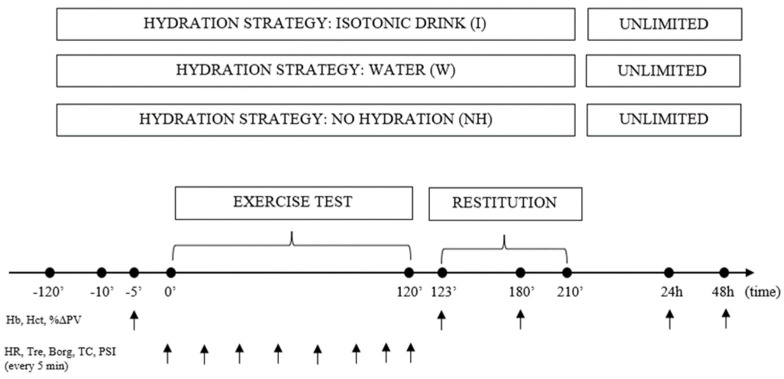
Diagram of one series of tests during the main part of the study including measurement points.

Prior to exercise (120 and 10 min before the test), the participants received an isotonic drink or water in accordance with the standards of the American College of Sports Medicine [39] (Table 1, Figure 2).

#### 2.4.3. Rectal Temperature

Immediately prior to exercise, as well as during the test, the rectal temperature (T_re_) of the participants was recorded continuously with an MRV-A electrothermometer (Ellab, 2610 Rødovre. Series CTD85-M 962300 03. Krondalvej, Denmark). The thermocouples used to measure T_re_ at a depth of 15 cm were each time covered with a disposable sterile sheath in accordance with the instructions of Ellab; prior to each test, the sensors were sterilized in an autoclave at a temperature of 120 °C, and then chemically disinfected.

#### 2.4.4. Thermal Load

To determine the effect of thermal load during exercise, an index based on the measurements of heart rate and T_re_ was applied [40].

The physical strain index (PSI), reflecting the physiological load expressed on a 10-grade numerical scale, is indicated by the following formula:

PSI = 5 · (T_ret_ − T_re0_) · (39.5 − T_re0_)^−1^ + 5 · (HR_t_ − HR_0_) · (180 − HR_0_)^−1^

where

- T_ret_ and HR_t_ are the values of rectal temperature and heart rate determined at any time during the exercise,
- T_re0_ and HR_0_ are the baseline values, determined before the test.

#### 2.4.5. Workload Assessment

The subjective perception of workload was assessed using the Borg scale. The respondents rated their perceptions concerning the severity of workload before and during the exercise, at 5 min intervals.

### 2.5. Statistical Analysis

The statistical analysis was performed using the SAS 9.3 software (SAS Institute Inc., Cary, NC, USA). The hypotheses regarding the effects of the hydration strategy, the test order, and their interaction (HS, Trial, HS × Trial) on the mean values of the investigated indices were tested using the analysis of covariance (ANCOVA) for repeated measurements. This was measured at intervals before, and at 40, 80, and 115 min during three consecutive exercise tests using three different hydration strategies. The generalized linear mixed model (SAS MIXED procedure) with the restricted maximum likelihood estimation method was applied. The likelihood ratio test served to compare model fit with different variance structures. Mean values were estimated with the least squares method and then, if necessary, corrected with the Tukey–Kramer post hoc test. The applied model included one random variable (respondent’s identification number [ID]), as well as two independent variables (hydration strategy [HS], test order [Trial]) and their interactions. Appropriate transformations were used in the case of non-normality or heteroscedasticity of the values. A test probability level of *p* < 0.05 was assumed significant.

## 3. Results

As shown by the results, the mean body height of the participants was 177.3 ± 4.8 cm, with an average body mass of 74.5 ± 7.6 kg. The body mass index remained within the reference values. The examined males were very similar in age (Table 2).

The VO_2_ value was 49.7 ± 6.7 mL/min/kg. The maximum heart rate was 187.8 ± 6.7 beats/min, with a maximum respiratory minute ventilation of 132.8 ± 43.6 L/min. Detailed changes in the physiological indices during the graded test are provided in Table 2. The mean power generated by the men studied was 663.1 ± 72.0 W, with a peak power of 827.2 ± 98.0 W (Table 2).

### Dehydration Index

**Figure 3:**
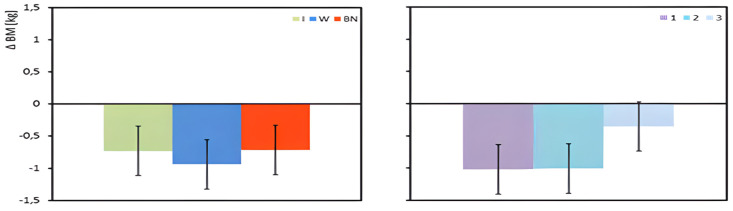
Change in body mass (∆BM) between pre-test and post-test state. Mean values estimated using the least squares method along with upper and lower confidence intervals. Measurements were taken from 12 men in three consecutive tests (1, 2, 3) using three hydration strategies (I—isotonic drink, W—water, BN—none).

Regardless of the applied hydration strategy, the participants’ mean body mass decreased under the influence of exercise. The largest body mass loss was recorded when using water (Table 3, Figure 3), with between-group differences not statistically significant (Table 4).

**Figure 4:**
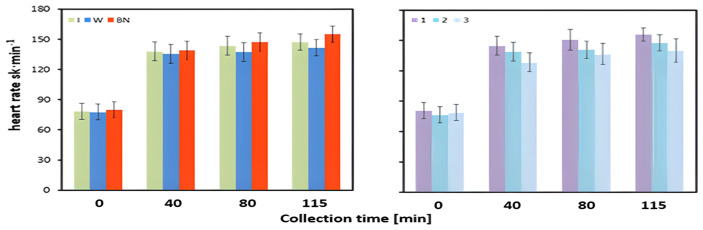
Heart rate. Mean values estimated using the least squares method along with upper and lower confidence intervals for four measurements (immediately before the test—preT, during the test—40, 80, 115 min of exertion) taken from 12 men in three consecutive tests (1, 2, 3) using three hydration strategies (I—isotonic drink, W—water, BN—none).

The mean heart rate measured before the test was 78.6 beats/min and did not differ significantly between hydration strategies, test order, or the interaction of their effects (Table 3, Figure 4).

The interpretation of the results after 80 min of exercise is complicated by the significant interaction of the effects of the hydration strategy and test order (Table 5). After 80 min of test 1, the heart rate was significantly higher for participants taking no hydration than for those taking isotonic drinks (*p* = 0.024) or water (*p* = 0.021) intake. In test 2, the highest heart rate value was recorded when an isotonic drink was used; this value was significantly higher than that for water intake (*p* = 0.012) but did not differ from no hydration (*p* = 0.714). A significantly higher heart rate was observed for no hydration than for water intake (*p* = 0.039). In test 3, the heart rate values were similar and did not differ significantly (*p* = 0.557) (Table 5).

**Figure 5:**
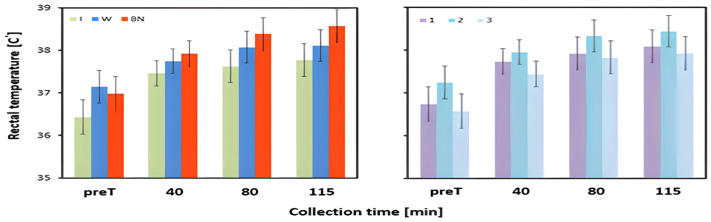
Rectal temperature; mean values estimated using the least squares method with upper and lower confidence intervals for four measurements (immediately before the test—preT, during the test—40, 80, 115 min of exertion) taken from 12 men in three consecutive tests (1, 2, 3) using three hydration strategies (I—isotonic drink, W—water, BN—none).

The hydration strategy and test order significantly influenced Tre in the measurements before the test and at 40 and 115 min of exercise. The interaction of the fixed factor effects had a statistically significant impact on the mean Tre values measured at 80 min (Table 3, Figure 5). For each measurement taken during exercise, the highest Tre values were recorded for no hydration (at 40, 80, and 115 min), and the lowest Tre values were noted for isotonic drink intake. With this strategy, Tre was the lowest also before the test (Table 4). Statistically significant differences for Tre were found between the use of an isotonic drink and no hydration in three measurements before the test (*p* = 0.03), at 40 min (*p* = 0.015), and 115 min (*p* = 0.002). Moreover, there were significant differences in Tre before the test between isotonic drink and water intake (*p* = 0.005), as well as at 115 min between water usage and no hydration (*p* = 0.036). The remaining differences were not significant (Table 3 and Table 4).

In test 1, the values of Tre were significantly lower at 80 min for isotonic drink intake than for water intake (*p* = 0.013) or no hydration (*p* = 0.001). In test 2, the highest Tre was also observed for no hydration and this value was significantly higher than that for water intake (*p* = 0.01). Hydration with an isotonic drink resulted in an average level of Tre. In test 3, the highest Tre was noted for water intake and the lowest one for isotonic drink intake. This difference was significant (*p* = 0.038). No hydration resulted in an average level of Tre, which was not significantly different than that for the isotonic drink or water intake subjects (Table 5).

**Figure 6:**
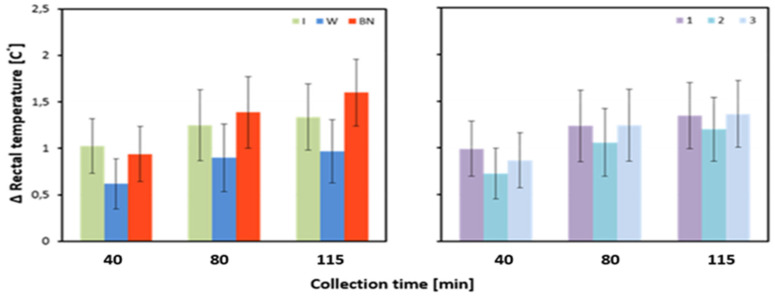
Change in average rectal temperature between pre-test and post-test state (∆Tre_avg3); mean values estimated using the least squares method with upper and lower confidence intervals; measurements taken from 12 men in three consecutive tests (1, 2, 3) using three hydration strategies (I—isotonic drink, W—water, BN—none).

The rise of Tre between baseline and the value at 40 min of exercise did not differ significantly between the hydration strategies. It is worth noting that at this stage of the experiment, the highest increase in temperature was recorded for hydration with an isotonic drink and the lowest with the use of water. A significant effect of the hydration strategy was observed for Tre changes between the status before the test and that at 80 and 115 min of effort. In both cases, the highest increase occurred for no hydration, and the lowest was found for the use of water. These differences were statistically significant, with the respective values of *p* = 0.026 and *p* = 0.001 (Table 3 and Table 4, Figure 6).

**Figure 7:**
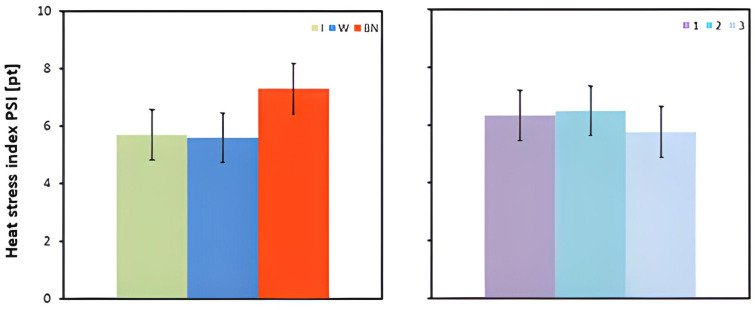
Heat stress index PSI; mean values estimated using the least squares method with upper and lower confidence intervals, measured in 12 men in three consecutive tests (1, 2, 3) using three hydration strategies (I—isotonic drink, W—water, BN—none).

The mean PSI values differed significantly between the hydration strategies. No hydration resulted in a higher PSI value than the use of an isotonic drink or water (*p* < 0.001) (Table 3 and Table 4, Figure 7).

**Figure 8:**
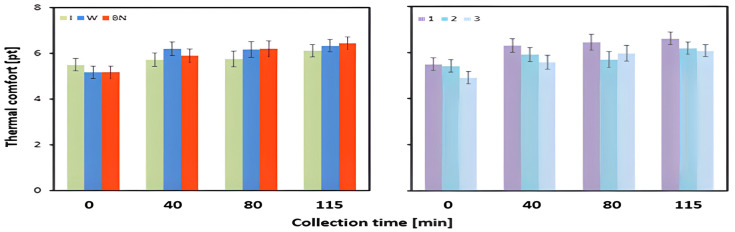
Thermal comfort; mean values estimated using the least squares method with upper and lower confidence intervals, for four measurements (TC_avg3) taken from 12 men (immediately before the test—0, and at 40, 80, and 115 min of exercise) in three consecutive tests (1, 2, 3) using three hydration strategies (I—isotonic drink, W—water, BN—none).

The mean values of the subjective assessment of thermal comfort differed significantly between the hydration strategies at 40 and 80 min of the test (Table 3). It should also be noted that throughout the duration of the exercise, at each stage, the use of an isotonic drink resulted in lower thermal comfort values, whereas no hydration and the intake of water were associated with higher thermal comfort (Table 3, Figure 8).

After 40 min of the test, the use of an isotonic drink resulted in significantly lower values than for water intake (*p* = 0.002). Furthermore, the use of water was associated with significantly higher values than no hydration (*p* = 0.038). After 80 min of the test, the consumption of an isotonic drink yielded significantly lower values than water use (*p* = 0.045). The use of an isotonic drink also resulted in significantly lower values than for no hydration (*p* = 0.032). At the end of the stress test, at 115 min, the highest values were recorded for no hydration, medium values for water use, and the lowest values for isotonic drink intake; these differences, however, were not significant (Table 3 and Table 4).

**Figure 9:**
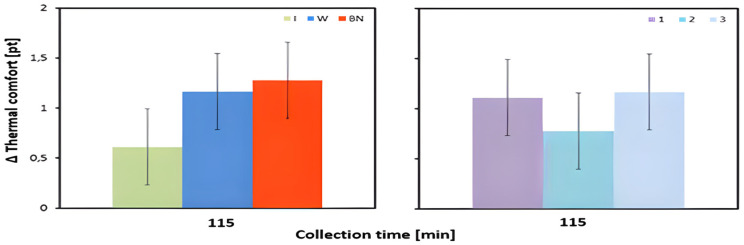
Thermal comfort; mean values estimated using the least squares method along with upper and lower confidence intervals, for four measurements (TC_avg3) taken from 12 men (test 115 min of exercise) in three consecutive tests (1, 2, 3) using three hydration strategies (I—isotonic drink, W—water, BN—none).

The mean values of the changes in the subjective perception of thermal comfort between the pre-test status and at 115 min of exercise were significantly influenced by the interaction of hydration strategy and test order (Table 3, Figure 9). It should be emphasized that in each of the three tests, the lowest values of change in thermal comfort levels occurred for isotonic drink use (Table 5).

**Figure 10:**
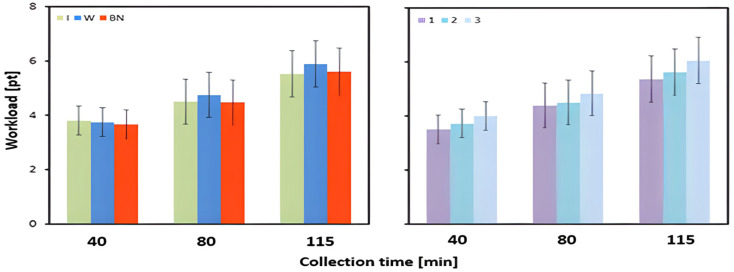
Workload (Borg scale); mean values estimated by the least squares method with upper and lower confidence intervals for three measurements (SBorg_avg3) performed at 40, 80, and 115 min of exercise in 12 men during three consecutive tests (1, 2, 3) using three hydration strategies (I—isotonic drink, W—water, BN—none).

The mean subjective workload values (Borg scale) did not differ significantly between the hydration strategies (Table 3 and Table 4, Figure 10).

**Figure 11:**
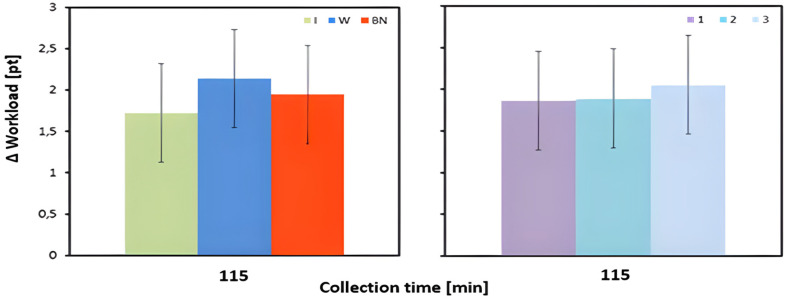
Change in the subjective perception of workload (Borg scale); mean values estimated by least squares method with upper and lower confidence intervals, measured between preT value and 115 min (SBorg_avg3) of three consecutive tests (1, 2, 3) performed on 12 male participants using three hydration strategies (I—isotonic drink, W—water, BN—none).

The changes in the subjective perception of workload (Borg scale) were not significantly influenced by the hydration strategy. Regardless of the strategy, the level of subjective load increased during exercise. A slight increase was observed for water intake compared to no hydration, and the lowest increase was found for isotonic drinks (Table 3 and Table 4, Figure 11).

## 4. Discussion

The effect on the athlete’s body of physical stimuli of different intensities, range, duration, and hydration strategies at room temperature has been the subject of research by many scientists while the knowledge gained empirically enables sports practitioners to optimize athletes’ preparation for competition. Slightly less information relates to the same measures performed at a thermoneutral temperature, close to the weighted average skin temperature. In the opinion of the authors of the present study, the mere comparison of the effect of different hydration strategies under thermally different conditions on changes in physiological-biochemical indices seems to be a novel approach to the problem. There are numerous reports in the literature of the negative effects of elevated ambient temperature on aerobic capacity [41,42,43]. Based on previous research, it could be assumed that the main cause of the decreased performance at elevated ambient temperatures is a state of dehydration, associated with an increase in thermoregulatory processes, mainly sweating [14,44,45].

In this study, regardless of the applied hydration strategy, the mean body mass of participants decreased with exercise. The largest body mass loss was recorded when using water; the between-group differences were not statistically significant. Physical exercise, including exercise undertaken at elevated ambient temperatures, is accompanied by an increased rate of metabolic processes and endogenous heat production [3,46]. Prolonged endogenous and exogenous thermal stress leads to increased dehydration, as observed in our research, regardless of the hydration strategy. This was mainly manifested by a weight loss of 0.8 kg on average. The largest decrease was reported for the use of water, with a mean of 0.94 kg. The change in body mass indicated in this study, induced by exercise at elevated ambient temperatures, was probably related to the loss of body water due to increased sweat production. This may have resulted in changes in the effective molality of body fluids and increased water transfer from the intracellular to extracellular space and then to the sweat glands, a mechanism also confirmed in previous studies [46,47,48]. Pilch et al. [49] revealed that the weight loss induced by heat exposure in a Finnish sauna in athletes was 0.95 kg. The body mass changes observed in the present study could have resulted from increased total body water (44.1 ± 4.4 kg) and decreased body fat mass (13.3 ± 2.4 kg). Caldwell et al. [50] and Costill et al. [51] argue that the cause of dehydration can affect how water is distributed in particular body spaces. As shown by Kozlowski and Saltin [52], dehydration of a purely thermal nature causes fluid loss from the extracellular space and only secondarily from the intracellular space, while dehydration induced by exercise leads to an equal reduction in water volume from both spaces.

In this study, statistically significant differences for Tre were found between the use of an isotonic drink and no hydration at three measurement stages: before the test, at 40 min, and 115 min. Moreover, there were significant differences in Tre before the test between isotonic drink and water intake, as well as at 115 min between water usage and no hydration. The highest increase in Tre temperature was observed for no hydration and the lowest for the use of water. The internal body temperature rises with the production of endogenous heat induced by physical exercise. Increases in internal temperature can be exacerbated by limited thermoregulatory capacity due to high temperature and humidity. Such ambient conditions impair most of the effective ways of dissipating heat from the body. The results of our study are corroborated by previous research [53,54], which demonstrated that, regardless of the hydration strategy, especially no hydration, Tre increases with the duration of exercise and can exceed 38 °C or even 40 °C. It is worth noting that dehydration begins to impair aerobic capacity when the skin temperature is above 27 °C, with each additional increase in skin temperature by 1 °C further reducing the body performance by almost 2% [12]. Coso et al. [53] compared the effects of hydration with popular isotonic drinks and water, as well as a no-hydration strategy in male athletes and non-athletes. The participants performed a 120 min stress test on a cycle ergometer at an elevated temperature, relatively low humidity, and fixed airflow rate (36 °C, 29%, 1.9 m/s). In all experimental groups, Tre gradually increased during exercise and reached values above 38 °C. These results are in line with those obtained in our research. In all groups, Tre reached a value of 38 °C. The slightly lower mean Tre can be explained by the fact that the participants in the present experiment pedaled at a constant physical load of 53% VO_2_max, which is 10% lower than in the study by Coso et al. [53]. It has been proven that compared with consuming an isotonic drink or water during exercise, no hydration resulted in significantly higher Tre values at the end of the exercise (*p* < 0.05) [53]. Szubert et al. [55], conducting a study on methods for determination of the relative exercise load of the human body, showed that, at medium and high exercise intensities, Tre was 37.95 °C and 38.58 °C, respectively. The results correlate with those in our research. Based on an analysis of the available literature and the results of our research, it can be assumed that in the case of dehydration associated with workload and high ambient temperature, the use of any hydration, such as with an isotonic drink or water, is crucial to compensate for the increasing thermal load. In particular, the use of an isotonic drink has shown high effectiveness in lowering Tre, as evidenced by the results obtained at 115 min of exercise. The authors of the previously cited research also pointed out that it was only from the 70th minute of exercise that differences in Tre were noted between the groups. Furthermore, they reported that water vs. isotonic drink intake had no substantial effect on the internal body temperature. Similar conclusions can be drawn when analyzing the results of our study. Significant differences in mean Tre could only be seen between any hydration and no hydration. These differences increased with exercise duration and were statistically significant only at the end of the exercise. It is noteworthy that Tre in each measurement during exercise with no hydration reached the highest values (40, 80, and 115 min), and, for hydration with an isotonic drink it was the lowest in each of the four measurements (before the test and at 40, 80, and 115 min). Furthermore, when analyzing the increase in temperature during the exercise, it was observed that the smallest increase in Tre occurred when applying water.

Heart rate is among the easiest to monitor and also the most researched method for evaluating exercise intensity. Working muscle cells require significant amounts of oxygen and energy substrates, while increased thermoregulation necessitates the transfer of blood supplies from the body interior to the periphery. This involves accelerating the heart rate. Thus, the increasing thermal load resulting from dehydration and impairment in the efficiency of the thermoregulatory processes should be manifested by a rise in heart rate. In the light of the research, it seems reasonable to consider this indicator as a reliable source of information about the severity of stress induced by exercise at elevated temperatures and humidity on the human body [29,41,42,43,56].

In a study of the effects of isotonic beverage intake (before and during exercise) on cardiovascular responses in trained and untrained men, Coso et al. [53] demonstrated that the use of such a beverage, as opposed to no hydration, significantly reduced exercise-induced heart rate. These results are consistent with those obtained in our research. In this study, both hydration with an isotonic drink and water resulted in the heart rate, HR, measured at 40 and 115 min of exercise being lower than in the absence of hydration; with a statistically significant difference in heart rate between water use and no hydration at 115 min of exercise. It can be presumed that at this stage of the exercise, the body could not cope with the increasing thermal load caused by effort-related and thermal dehydration. Furthermore, after 80 min of exercise, the interaction of the effects of the hydration strategy and the test order was significant, so it was reasonable to consider each series of tests as separate events. Nevertheless, the lowest heart rate values in all three series were recorded when an isotonic drink or water was used, which may confirm the effectiveness of their intake during such exercise. At this stage of the experiment (80 min), however, the differences between the three strategies were not significant. It should be noted that the application of water or isotonic drink did not induce significant differences in mean heart rate throughout the exercise. Similar conclusions can be drawn when analyzing the changes in heart rate during stress tests. Significant differences in heart rate did not occur until the 115th minute of exercise between no hydration and water intake, which caused the smallest increase in heart rate.

Based on the results of the present study, one can assume that the reduction in exercise heart rate was not dependent on the composition of the hydration drink, although blood glucose levels were not analyzed. The lower heart rate was most likely due to rehydration, which enabled more efficient thermoregulation. Such a finding is consistent with previous reports, in which the authors suggested that in the case of exercise performance being reduced due to hyperthermia induced by exercise at elevated ambient temperatures, beverages with a carbohydrate content of 6–8% may even exacerbate the cardiovascular burden [57,58]. In hyperthermia, a decrease in muscle glycogen stores and blood glucose levels is not the main factor limiting the body’s exercise capacity [59]. This is supported by the results of our study, which revealed that the use of water before, during, and after exercise was at least as effective in reducing heart rate as the use of an isotonic drink. Thus, based on the analysis of the two physiological body load indices (heart rate and Tre), it is not possible to conclude which type of hydration is more effective. Recently, PSI has become a popular indicator of physical load on the body used by many authors [57,58,60,61]. Numerous researchers have focused on how different volumes of fluid replenishment affect the body’s exercise capacity. Thus, the aim of most studies was to determine the optimal level of rehydration of the body during exercise rather than, as in the case of the present study, to investigate the effectiveness of different rehydration strategies.

Pompermayer et al. [62] conducted an experiment in which 10 untrained men performed exercise twice with a load of approximately 100 W until they lost 2% of their body mass or reached a Tre exceeding 39.5 °C. The test was carried out at an elevated ambient temperature (37 °C) and moderate humidity (45%). A stress test with no hydration and with hydration controlled at 20 min intervals was used to cover (during exercise) 100% of the water losses resulting from thermoregulatory processes. The results of the study demonstrated that the assumed hydration strategy significantly reduced the body thermal load expressed in terms of PSI. This conclusion is also supported by the results presented in our research. With no hydration, the mean PSI value was significantly higher than for both isotonic drink or water intake. These results are very consistent with those presented above, which may indicate the high reliability of the PSI index. Our research also showed that the type of hydration had no significant effect on PSI. As with other indices, it can be assumed that the main problem of working under experimental conditions was dehydration and the resultant hyperthermia. Irrespective of the type of hydration, the above-mentioned inhibiting factors were compensated for by replenishing water losses.

In this study, the mean PSI values were significantly different across the hydration strategies; the non-hydration group showed a higher PSI compared to the water and isotonic drink groups. The stress on the body expressed by the objective index does not always correlate with the subjective perceptions of the person exposed to elevated temperatures and exertion. Therefore, the present study also used the subjective assessment of thermal comfort and workload [63,64]. 

The subjective assessment of thermal comfort was performed in accordance with the Ashare scale. The respondents indicated the best comfort and satisfaction with the thermal environment when using an isotonic drink. This was also manifested in the results of the change in the perception of thermal comfort during exercise. The smallest changes in the perception of thermal comfort were observed for isotonic drink hydration whereas the largest changes were found for no hydration. This may indicate a beneficial effect from the isotonic drink on mental resistance to thermal stress, which increased with the duration of the exercise.

A frequently used tool for assessing subjective perception of workload is the assessment of workload on the Borg scale, which was used in our study. The respondents did not present any significant differences in perceived workload with respect to various hydration strategies. The highest values were recorded when water was used, which may be due to the high sweating rate, made possible by this rehydration method. Profuse sweating may have influenced the subjective assessment of workload. This inference seems to be confirmed by an analysis of weight change during the stress test. It was observed that the hydration strategies did not have a statistically significant effect on its magnitude; nevertheless, a slightly greater decrease in body mass was recorded for water intake. Thus, the analysis of the aforementioned results, as well as the observations concerning Tre, heart rate, and PSI, show that both water and isotonic drink hydration have a beneficial effect on the thermoregulatory processes, but it is the isotonic drink that inhibits dehydration to a greater extent.

### Limitations of the Study

The main limitation of this study was the relatively small sample size since participants were elite athletes. Additionally, during the experiment, some participants failed to complete the entire research procedure.

## 5. Conclusions

The results of the study allowed the formulation of the following conclusions:

1. During sustained exercise at elevated ambient temperatures and humidity, the use of hydration with an isotonic drink or water significantly improved thermoregulatory processes compared to no hydration in the male participants.
2. The subjective perceptions of workload under experimental conditions did not differ significantly with the use of the different hydration strategies. However, isotonic drink intake reduced the degree of thermal discomfort in the men surveyed.

## Figures and Tables

**Table 1:** Hydration strategy incorporating a crossover design.

Group	Number of Participants	Hydration in Successive Tests		
		Test I	Test II	Test III
I-W-NH	2	I	W	NH
W-NH-I	2	W	NH	I
NH-I-W	2	NH	I	W
I-NH-W	2	I	NH	W
W-I-NH	2	W	I	NH
NH-W-I	2	NH	W	I

I—isotonic drink; W—water; NH—no hydration.

**Table 2:** The somatic indices of the men studied, and selected indices of aerobic capacity and anaerobic capacity.

Variable		Mean	SD
Age (years)		20.7	1.0
BH (cm)		177.3	4.8
BM (kg)		74.5	7.6
LBM (kg)		61.2	6.2
BFM (kg)		13.3	2.4
BFP (%)		17.8	2.2
TBW (kg)		44.1	4.4
BMI (kg/m^2^)		23.7	2.1
Max (min)	Maximum values	14.6	2.6
PP (W)		291.1	42.1
HRmax (beats/min)		187.8	6.7
VO_2_ (L/min)		3.7	0.7
VO_2_ (mL/min/kg)		49.7	6.7
V_E_max (L/min)		132.8	43.6
tVT2 (min)	VT2 values	7.6	2.0
HRVT2 (beats/min)		147.0	15.0
V_E_VT2 (L/min)		62.8	17.8
PVT2 (W)		185.6	33.9
VO_2_VT2 (L/min)		2.4	0.5
VO_2_VT2 (mL/min/kg)		32.7	6.1
BM (kg)	74.8		7.5
Ttest (s)	30.0		0.0
Load (kg)	6.2		0.6
MP (W)	663.1		72.0
MP (W/kg)	8.9		0.5
TW (kJ)	19.9		2.2
TW (J/kg)	266.3		16.1
PP (W)	827.2		98.0
PP (W/kg)	11.1		0.8
PDI (W/kg/s)	0.2		0.0
toPP (s)	4.3		0.5
tmPP (s)	4.2		1.4

BH—body height; BM—body mass; LBM—lean body mass; BFM—body fat mass; BFP—body fat percentage; TBW—total body water; BMI—body mass index, Maxt—maximum exercise time; PP—peak power; HRmax—maximum heart rate; VO2max—maximal oxygen uptake: globally (L/min) and in relation to body mass (mL/min/kg); VEmax—maximum respiratory minute ventilation; VT2—second ventilatory threshold; tVT2—time to obtain VT2; HRVT2—heart rate at VT2; VEVT2—respiratory minute ventilation at VT2; PVT2—power at VT2; VO2VT2—oxygen uptake at VT2: globally (L/min) and in relation to body mass (mL/min/kg); Ttest—test time; MP—mean power; TW—total work; PDI—power drop index; toPP—time to obtain peak power; tmPP—time to maintain peak power.

**Table 3:** Results of analysis of variance of body mass (before and after the stress test), heart rate, rectal temperature, heart rate change, rectal temperature change, physical strain index (before and after the stress test), perception of thermal comfort and Borg scale.

Variables			Hydration Strategy			Test Order			Interaction		
				(DFl = 3)			(DFl = 3)			(DFl = 3)	
			DFm	F	*p*	DFm	F	*p*	DFm	F	*p*
Body mass change (kg)			17.7	0.39	0.683	17.7	3.59	0.049	17.2	1.46	0.257
Heart rate	Time	preT	16.5	0.36	0.703	16.5	1.10	0.355	20.0	1.42	0.263
		40	16.2	0.54	0.595	16.2	11.47	0.001	20.3	1.55	0.225
		80	11.8	6.76	0.011	7.7	56.62	<0.001	8.4	10.03	0.003
		115	11.6	14.97	0.001	6.8	17.70	0.002	6.6	4.69	0.041
Rectal temperature	Time	preT	13.3	7.79	0.006	13.3	8.08	0.005	17.00	3.77	0.023
		40	13.4	5.46	0.018	13.4	7.90	0.005	17.00	3.98	0.019
		80	11.2	15.52	0.001	11.2	9.42	0.004	12.60	6.86	0.004
		115	11.6	10.55	0.002	11.6	5.33	0.023	15.40	3.30	0.039
Heart rate change	Time	40	16.3	0.15	0.862	16.3	9.81	0.002	18.9	1.98	0.140
		80	16.2	0.58	0.571	16.2	3.68	0.048	19.9	1.81	0.167
		115	16.1	3.79	0.045	16.1	5.54	0.015	19.4	1.47	0.249
Rectal temperature change	Time	40	15.9	3.39	0.059	15.9	1.28	0.305	21.5	1.80	0.165
		80	15.0	4.57	0.028	15.0	0.78	0.476	18.2	2.10	0.122
		115	14.3	10.62	0.002	14.3	0.83	0.457	16.6	3.84	0.022
Physical strain index			14.1	18.74	<0.0001	14.1	3.16	0.074	15.7	3.56	0.030
Perception of thermal comfort	Time	preT	12.6	2.78	0.100	12.6	7.47	0.007	27.0	2.29	0.085
		40	15.3	9.23	0.002	15.3	21.04	<0.001	19.7	2.46	0.079
		80	14.8	5.03	0.022	14.8	11.67	0.001	22.2	3.02	0.040
		115	16.3	2.33	0.129	16.3	6.27	0.010	26.9	3.47	0.021
Borg scale	Time	40	12.6	0.13	0.876	12.6	1.72	0.218	23.5	0.44	0.778
		80	13.3	0.38	0.691	13.3	0.87	0.442	20.0	1.59	0.215
		115	13.9	0.58	0.575	13.9	2.00	0.173	19.9	1.45	0.255

Significance (*p*) and F statistics with the number of degrees of freedom (DFl in the numerator, DFm in the denominator) for fixed effects (hydration strategy, test order, and their interaction). Level of significance: *p* < 0.05.

**Table 4:** Mean values of variables between baseline and post-exercise status in 3 consecutive tests (1, 2, 3) performed with 3 different hydration strategies.

Statistics				Hydration Strategy			Test Order		
				I	W	NH	1	2	3
Body mass change (kg)			LSM	−0.73	−0.94	−0.72	−1.02	−1.01	−0.35
			SE	0.19	0.19	0.19	0.19	0.19	0.19
			LCL	−1.11	−1.32	−1.10	−1.41	−1.39	−0.74
			UCL	−0.35	−0.56	−0.33	−0.64	−0.63	0.03
Rectal temperature	time	preT	LSM	36.4	37.1	37.0	36.7	37.2	36.6
			SE	0.2	0.2	0.2	0.2	0.2	0.2
			LCL	36.0	36.8	36.6	36.3	36.9	36.2
			UCL	36.8	37.5	37.4	37.1	37.6	37.0
		40	LSM	37.5	37.7	37.9	37.7	38.0	37.4
			SE	0.1	0.1	0.1	0.1	0.1	0.1
			LCL	37.2	37.5	37.6	37.4	37.7	37.1
			UCL	37.8	38.0	38.2	38.0	38.2	37.7
		80	LSM	37.6	38.1	38.4	37.9	38.3	37.8
			SE	0.2	0.2	0.2	0.2	0.2	0.2
			LCL	37.2	37.7	38.0	37.5	38.0	37.4
			UCL	38.0	38.4	38.8	38.3	38.7	38.2
		115	LSM	37.8	38.1	38.6	38.1	38.4	37.9
			SE	0.2	0.2	0.2	0.2	0.2	0.2
			LCL	37.4	37.7	38.2	37.7	38.1	37.5
			UCL	38.2	38.5	39.0	38.5	38.8	38.3
Heart rate change	time	40	LSM	59.7	57.8	59.0	64.2	62.6	49.7
			SE	5.5	5.5	5.5	5.5	5.5	5.5
			LCL	47.9	46.0	47.2	52.4	50.8	37.9
			UCL	71.5	69.6	70.8	76.0	74.4	61.5
		80	LSM	64.3	61.5	66.3	70.0	64.3	57.9
			SE	5.9	5.9	5.9	5.9	5.9	5.9
			LCL	51.8	49.0	53.8	57.5	51.8	45.3
			UCL	76.8	74.0	78.8	82.5	76.8	70.4
		115	LSM	69.8	63.0	74.9	75.3	71.2	61.3
			SE	5.9	5.9	5.9	5.9	5.9	5.9
			LCL	57.1	50.3	62.2	62.6	58.5	48.6
			UCL	82.5	75.7	87.6	87.9	83.9	74.0
Rectal temperature change	time	40	LSM	1.0	0.6	0.9	1.0	0.7	0.9
			SE	0.1	0.1	0.1	0.1	0.1	0.1
			LCL	0.7	0.3	0.6	0.7	0.5	0.6
			UCL	1.3	0.9	1.2	1.3	1.0	1.2
		80	LSM	1.2	0.9	1.4	1.2	1.1	1.2
			SE	0.2	0.2	0.2	0.2	0.2	0.2
			LCL	0.9	0.5	1.0	0.9	0.7	0.9
			UCL	1.6	1.3	1.8	1.6	1.4	1.6
		115	LSM	1.3	1.0	1.6	1.3	1.2	1.4
			SE	0.2	0.2	0.2	0.2	0.2	0.2
			LCL	1.0	0.6	1.2	1.0	0.9	1.0
			UCL	1.7	1.3	2.0	1.7	1.5	1.7
Physical strain index			LSM	5.71	5.59	7.30	6.33	6.50	5.77
			SE	0.41	0.40	0.41	0.41	0.40	0.41
			LCL	4.83	4.74	6.42	5.45	5.65	4.89
			UCL	6.58	6.44	8.18	7.21	7.35	6.64
Thermal comfort	time	preT	LSM	5.50	5.17	5.17	5.50	5.42	4.92
			SE	0.13	0.13	0.13	0.13	0.13	0.13
			LCL	5.23	4.89	4.89	5.23	5.14	4.64
			UCL	5.77	5.44	5.44	5.77	5.69	5.19
		40	LSM	5.72	6.19	5.89	6.31	5.92	5.58
			SE	0.14	0.14	0.14	0.14	0.14	0.14
			LCL	5.42	5.90	5.59	6.01	5.62	5.29
			UCL	6.02	6.49	6.19	6.60	6.21	5.88
		80	LSM	5.75	6.17	6.19	6.44	5.69	5.97
			SE	0.16	0.16	0.16	0.16	0.16	0.16
			LCL	5.41	5.82	5.85	6.10	5.35	5.63
			UCL	6.09	6.51	6.54	6.79	6.04	6.31
	time	115	LSM	6.11	6.33	6.44	6.61	6.19	6.08
			SE	0.13	0.13	0.13	0.13	0.13	0.13
			LCL	5.85	6.07	6.18	6.35	5.93	5.82
			UCL	6.38	6.60	6.71	6.88	6.46	6.35
Workload (Borg scale)		40	LSM	3.81	3.75	3.67	3.50	3.72	4.00
			SE	0.25	0.25	0.25	0.25	0.25	0.25
			LCL	3.28	3.22	3.14	2.97	3.20	3.47
			UCL	4.33	4.28	4.19	4.03	4.25	4.53
		80	LSM	4.50	4.75	4.47	4.39	4.50	4.83
			SE	0.39	0.39	0.39	0.39	0.39	0.39
			LCL	3.68	3.93	3.65	3.57	3.68	4.01
			UCL	5.32	5.57	5.29	5.21	5.32	5.66
		115	LSM	5.53	5.89	5.61	5.36	5.61	6.06
			SE	0.40	0.40	0.40	0.40	0.40	0.40
			LCL	4.67	5.03	4.76	4.51	4.76	5.20
			UCL	6.38	6.74	6.47	6.22	6.47	6.91
Change in Borg scale		115	LSM	1.7	2.1	1.9	1.9	1.9	2.1
			SE	0.3	0.3	0.3	0.3	0.3	0.3
			LCL	1.1	1.5	1.4	1.3	1.3	1.5
			UCL	2.3	2.7	2.5	2.5	2.5	2.6

I—isotonic drink; W—water; NH—no hydration. preT—Pre-test, SE—standard errors, LSM—marginal means, LCI—lower and UCI—upper confidence intervals.

**Table 5:** Results for the values averaged over 3 consecutive measurements of heart rate and rectal temperature at 80 min, and thermal comfort change at 115 min of the 3 consecutive stress tests (1, 2, 3) performed with 3 different hydration strategies.

Variables		HR	Test Order	1			2			3		
			Strategy	I	W	NH	I	W	NH	I	W	NH
Heart rate	time	80	LSM	146.3	146.0	159.5	150.6	126.3	145.5	133.6	139.7	136.6
			SE	5.1	5.1	5.1	5.3	5.3	5.3	5.7	5.7	5.7
Rectal temperature	time	80	LSM	37.1	38.2	38.5	38.3	37.9	38.8	37.5	38.2	37.8
			SE	0.3	0.2	0.2	0.2	0.2	0.2	0.2	0.2	0.3
Thermal comfort change	time	115	LSM	0.18	1.89	1.26	0.76	0.06	1.52	0.89	1.56	1.05
			SE	0.31	0.31	0.31	0.31	0.31	0.31	0.31	0.31	0.31

I—isotonic drink; W—water; NH—no hydration; LSM—least squares mean; SE—standard error.

## Data Availability

All data are included in the manuscript.

## References

[1] Rodriguez N.R., DiMarco N.M., Langley S. (2000). Position of the American Dietetic Association, Dietitians of Canada, and the American College of Sports Medicine. J. Am. Diet. Assoc..

[2] Maughan R.J., Shirreffs S.M. (2010). Development of Hydration Strategies to Optimize Performance for Athletes in High-Intensity Sports and in Sports with Repeated Intense Efforts: Development of Hydration Strategies to Optimize Performance for Athletes. Scand. J. Med. Sci. Sports.

[3] Pokora I., Sadowska-Krępa E., Zając-Gawlak I., Pelclova J. (2015). Body Composition and Hydration Status in Young Elderly Women after 6 Weeks’ Monavie Juice Supplementation. AUC Kinanthropologica.

[4] Belval L.N., Hosokawa Y., Casa D.J., Adams W.M., Armstrong L.E., Baker L.B., Burke L., Cheuvront S., Chiampas G., González-Alonso J. (2019). Practical Hydration Solutions for Sports. Nutrients.

[5] Kenefick R.W., Cheuvront S.N. (2012). Hydration for Recreational Sport and Physical Activity. Nutr. Rev..

[6] Fitting J.-W. (2015). From Breathing to Respiration. Respiration.

[7] Périard J.D., Eijsvogels T.M.H., Daanen H.A.M. (2021). Exercise under Heat Stress: Thermoregulation, Hydration, Performance Implications, and Mitigation Strategies. Physiol. Rev..

[8] Sawka M.N., Burke L.M., Eichner E.R., Maughan R.J., Montain S.J., Stachenfeld N.S., American College of Sports Medicine (2007). Exercise and Fluid Replacement. Med. Sci. Sports Exerc..

[9] Shirreffs S.M., Sawka M.N. (2011). Fluid and Electrolyte Needs for Training, Competition, and Recovery. J. Sports Sci..

[10] Shirreffs S.M. (2005). The Importance of Good Hydration for Work and Exercise Performance. Nutr. Rev..

[11] Birch K., Gromadzka-Ostrowska J., MacLaren D., George K. (2012). Fizjologia Sportu.

[12] Bean A., Wojtczak E. (2014). Żywienie w Sporcie: Kompletny Przewodnik.

[13] Cheuvront S.N., Carter R., Montain S.J., Sawka M.N. (2004). Daily Body Mass Variability and Stability in Active Men Undergoing Exercise-Heat Stress. Int. J. Sport Nutr. Exerc. Metab..

[14] Deshayes T.A., Pancrate T., Goulet E.D.B. (2022). Impact of Dehydration on Perceived Exertion during Endurance Exercise: A Systematic Review with Meta-Analysis. J. Exerc. Sci. Fit..

[15] McDermott B.P., Anderson S.A., Armstrong L.E., Casa D.J., Cheuvront S.N., Cooper L., Kenney W.L., O’Connor F.G., Roberts W.O. (2017). National Athletic Trainers’ Association Position Statement: Fluid Replacement for the Physically Active. J. Athl. Train..

[16] Pitts G.C., Johnson R.E., Consolazio F.C. (1944). Work in the Heat as Affected by Intake of Water, Salt, and Glucose. Am. J. Phys..

[17] Maughan R.J., Shirreffs S.M. (2010). Dehydration and Rehydration in Competative Sport: Dehydration and Rehydration. Scand. J. Med. Sci. Sports.

[18] Cheuvront S.N., Kenefick R.W., Sollanek K.J., Ely B.R., Sawka M.N. (2013). Water-Deficit Equation: Systematic Analysis and Improvement. Am. J. Clin. Nutr..

[19] Sawka M.N., Cheuvront S.N., Kenefick R.W. (2012). High Skin Temperature and Hypohydration Impair Aerobic Performance: Skin Temperature, Hypohydration and Performance. Exp. Physiol..

[20] Nybo L., Jensen T., Nielsen B., González-Alonso J. (2001). Effects of Marked Hyperthermia with and without Dehydration onV’ o _2_ Kinetics during Intense Exercise. J. Appl. Physiol..

[21] Sawka M.N., Coyle E.F. (1999). Influence of Body Water and Blood Volume on Thermoregulation and Exercise Performance in the Heat. Exerc. Sport Sci. Rev..

[22] Sansone J.E., Guyer M.S., Mullin E.M., Thompson B. (2022). Fluid Restriction Dehydration Increase Core Temperature during Endurance Exercise Compared to Exercise Induced Dehydration. Int. J. Exerc. Sci..

[23] Wendt D., van Loon L.J.C., van Marken Lichtenbelt W.D. (2007). Thermoregulation during Exercise in the Heat: Strategies for Maintaining Health and Performance. Sports Med..

[24] EFSA Panel on Dietetic Products, Nutrition, and Allergies (NDA) (2010). Scientific Opinion on Dietary Reference Values for Water. EFS2.

[25] Nose H., Mack G.W., Shi X.R., Nadel E.R. (1988). Role of Osmolality and Plasma Volume during Rehydration in Humans. J. Appl. Physiol..

[26] Goodman S.P.J., Moreland A.T., Marino F.E. (2019). The Effect of Active Hypohydration on Cognitive Function: A Systematic Review and Meta-Analysis. Physiol. Behav..

[27] Baker L.B. (2017). Sweating Rate and Sweat Sodium Concentration in Athletes: A Review of Methodology and Intra/Interindividual Variability. Sports Med..

[28] Maughan R.J., Shirreffs S.M. (2008). Development of Individual Hydration Strategies for Athletes. Int. J. Sport Nutr. Exerc. Metab..

[29] Trangmar S.J., Chiesa S.T., Stock C.G., Kalsi K.K., Secher N.H., González-Alonso J. (2014). Dehydration Affects Cerebral Blood Flow but Not Its Metabolic Rate for Oxygen during Maximal Exercise in Trained Humans. J Physiol.

[30] Vitale K., Getzin A. (2019). Nutrition and Supplement Update for the Endurance Athlete: Review and Recommendations. Nutrients.

[31] Truong P., Millet G., Gojanovic B. (2018). Perceptually Regulated Exercise Test Allows Determination of V’O2max and Ventilatory Threshold but Not Respiratory Compensation Point In Trained Runners. Int. J. Sports Med..

[32] Binder R.K., Wonisch M., Corra U., Cohen-Solal A., Vanhees L., Saner H., Schmid J.-P. (2008). Methodological Approach to the First and Second Lactate Threshold in Incremental Cardiopulmonary Exercise Testing. Eur. J. Cardiovasc. Prev. Rehabil..

[33] Bar-Or O. (1987). The Wingate Anaerobic Test: An Update on Methodology, Reliability and Validity. Sports Med..

[34] Dotan R., Bar-Or O. (1983). Load Optimization for the Wingate Anaerobic Test. Europ. J. Appl. Physiol..

[35] Driss T., Vandewalle H. (2013). The Measurement of Maximal (Anaerobic) Power Output on a Cycle Ergometer: A Critical Review. BioMed Res. Int..

[36] Faiss R., Léger B., Vesin J.-M., Fournier P.-E., Eggel Y., Dériaz O., Millet G.P. (2013). Significant Molecular and Systemic Adaptations after Repeated Sprint Training in Hypoxia. PLoS ONE.

[37] Dill D.B., Costill D.L. (1974). Calculation of Percentage Changes in Volumes of Blood, Plasma, and Red Cells in Dehydration. J. Appl. Physiol..

[38] Harrison M.H., Graveney M.J., Cochrane L.A. (1982). Some Sources of Error in the Calculation of Relative Change in Plasma Volume. Europ. J. Appl. Physiol..

[39] Convertino V.A., Armstrong L.E., Coyle E.F., Mack G.W., Sawka M.N., Senay L.C., Sherman W.M. (1996). ACSM Position Stand: Exercise and Fluid Replacement. Med. Amp Sci. Sports Amp Exerc..

[40] Moran D.S., Shitzer A., Pandolf K.B. (1998). A Physiological Strain Index to Evaluate Heat Stress. Am. J. Physiol. -Regul. Integr. Comp. Physiol..

[41] Nybo L., Rasmussen P., Sawka M.N., Terjung R. (2014). Performance in the Heat—Physiological Factors of Importance for Hyperthermia-Induced Fatigue. Comprehensive Physiology.

[42] Nassis G.P., Brito J., Dvorak J., Chalabi H., Racinais S. (2015). The Association of Environmental Heat Stress with Performance: Analysis of the 2014 FIFA World Cup Brazil. Br. J. Sports Med..

[43] Racinais S., Périard J.D., Karlsen A., Nybo L. (2015). Effect of Heat and Heat Acclimatization on Cycling Time Trial Performance and Pacing. Med. Sci. Sports Exerc..

[44] Cheuvront S.N., Kenefick R.W., Terjung R. (2014). Dehydration: Physiology, Assessment, and Performance Effects. Comprehensive Physiology.

[45] Merry T.L., Ainslie P.N., Cotter J.D. (2010). Effects of Aerobic Fitness on Hypohydration-Induced Physiological Strain and Exercise Impairment. Acta Physiol..

[46] Pokora I. (2006). Heat Stress Responses in Men after Ingestion of a Low-Sodium Diet. New Med..

[47] Łaszczyńska J., Piotrowski M. (2007). Ocena wpływu odwodnienia termicznego na zdolność rozwijania siły mięśni antygrawitacyjnych. Pol. Przegląd Med. Lot..

[48] Thomas D.R., Cote T.R., Lawhorne L., Levenson S.A., Rubenstein L.Z., Smith D.A., Stefanacci R.G., Tangalos E.G., Morley J.E. (2008). Understanding Clinical Dehydration and Its Treatment. J. Am. Med. Dir. Assoc..

[49] Pilch W. (2009). Wpływ Podwyższenia Temperatury Ciała na Zmiany Wybranych Wskaźników Fizjologicznych i Status Antyoksydacyjny Osocza Biegaczy Długodystansowych oraz Mężczyzn Nietrenujących.

[50] Caldwell J.E., Ahonen E., Nousiainen U. (1984). Differential Effects of Sauna-, Diuretic-, and Exercise-Induced Hypohydration. J. Appl. Physiol..

[51] Costill D., Coté R., Fink W., Van Handel P. (1981). Muscle Water and Electrolyte Distribution during Prolonged Exercise. Int. J. Sports Med..

[52] Kozlowski S., Saltin B. (1964). Effect of Sweat Loss on Body Fluids. J. Appl. Physiol..

[53] Coso J.D., Estevez E., Baquero R.A., Mora-Rodriguez R. (2008). Anaerobic Performance When Rehydrating with Water or Commercially Available Sports Drinks during Prolonged Exercise in the Heat. Appl. Physiol. Nutr. Metab..

[54] Montain S.J., Coyle E.F. (1992). Influence of Graded Dehydration on Hyperthermia and Cardiovascular Drift during Exercise. J. Appl. Physiol..

[55] Szubert J., Szubert S., Koszada-Włodarczyk W., Bortkiewicz A. (2014). New met hods for dete rmining the relative load due to physical eff ort of the human body. Med. Pr..

[56] Cheuvront S.N., Haymes E.M. (2001). Thermoregulation and Marathon Running: Biological and Environmental Influences. Sports Med..

[57] Sanchis-Gomar F., Lucia A., Yvert T., Ruiz-Casado A., Pareja-Galeano H., Santos-Lozano A., Fiuza-Luces C., Garatachea N., Lippi G., Bouchard C. (2015). Physical Inactivity and Low Fitness Deserve More Attention to Alter Cancer Risk and Prognosis. Cancer Prev. Res..

[58] Maxwell N.S., Mackenzie R.W.A., Bishop D. (2009). Influence of Hypohydration on Intermittent Sprint Performance in the Heat. Int. J. Sports Physiol. Perform..

[59] Hargreaves M. (1996). Physiologlcal Benefits of Fluid and Energy Replacement during Exercise. Aust. J. Nutr. Diet..

[60] Richardson A., Watt P., Maxwell N. (2009). The Effect of Hypohydration Severity on the Physiological, Psychological and Renal Hormonal Responses to Hypoxic Exercise. Eur. J. Appl. Physiol..

[61] Pilch W., Szyguła Z., Palka T., Pilch P., Cison T., Wiecha S., Tota Ł. (2014). Comparison of physiological reactions and physiological strain in healthy men under heat stress in dry and steam heat saunas. Biol. Sport.

[62] Pompermayer M.G., Rodrigues R., Baroni B.M., Lupion R.D.O., Meyer F., Vaz M.A. (2014). Reidratação Durante Exercício No Calor Reduz o Índice de Esforço Fisiológico Em Adultos Saudáveis. Rev. Bras. Cineantropom. Desempenho Hum..

[63] Armstrong L.E., Ganio M.S., Casa D.J., Lee E.C., McDermott B.P., Klau J.F., Jimenez L., Le Bellego L., Chevillotte E., Lieberman H.R. (2012). Mild Dehydration Affects Mood in Healthy Young Women. J. Nutr..

[64] Stachenfeld N.S., Leone C.A., Mitchell E.S., Freese E., Harkness L. (2018). Water Intake Reverses Dehydration Associated Impaired Executive Function in Healthy Young Women. Physiol. Behav..

